# FT-IR spectroscopy analysis of HF-treated mineral soils, a direct approach for deciphering organo-mineral interactions

**DOI:** 10.1016/j.mex.2023.102088

**Published:** 2023-02-23

**Authors:** A. Spence, T. Wallace, E. Barracks

**Affiliations:** International Centre for Environmental and Nuclear Sciences, University of the West Indies, Mona, Kingston 7, Jamaica

**Keywords:** HF-treatment, Infrared spectroscopy, Organo-mineral interactions, Soil minerals, Soil organic matter, Soil organic carbon, HF-treatment of mineral soils for FT-IR analysis

## Abstract

Soil organic matter (SOM) constitutes roughly 60% organic carbon (OC) and therefore plays a crucial role in regulating global climate. However, our understanding of the long-term dynamics of the soil carbon pool remains constrained by limitations in analytical approaches capable of providing high resolution molecular-level information from arguably the most complex biomaterial on the planet. In this contribution, we combine hydrofluoric acid (HF) treatment with a spectroscopic approach as a strategy to provide refined molecular-level information on the interactions between soil minerals and SOM. Critically, we have not seen the use of this combined approach anywhere in the literature and strongly believe that it could allow us to improve our overall understanding to the mechanisms and pathways that regulate SOM transformation. Results clearly illustrates which organic structures are preferentially adsorbed to soil minerals and are likely to be protected from degradation, as well as spatial co-variations of SOM with specific mineral components such as Al^3+^, Si^4+^ and dibasic cations such as Mg^2+^as a function of their importance in the interaction process.

• Soil samples were collected from different land-use types in rural farming communities of the Upper Rio Grande Valley.
• Samples were oven dried, disaggregated, sieved, treated with 10% HF, rinsed and oven dried.
• Oven dried samples were subjected to Mid–infrared (4000–400 cm^−1^), XRD and ED-XRF analyses.

## Graphical abstract

**Figure:**
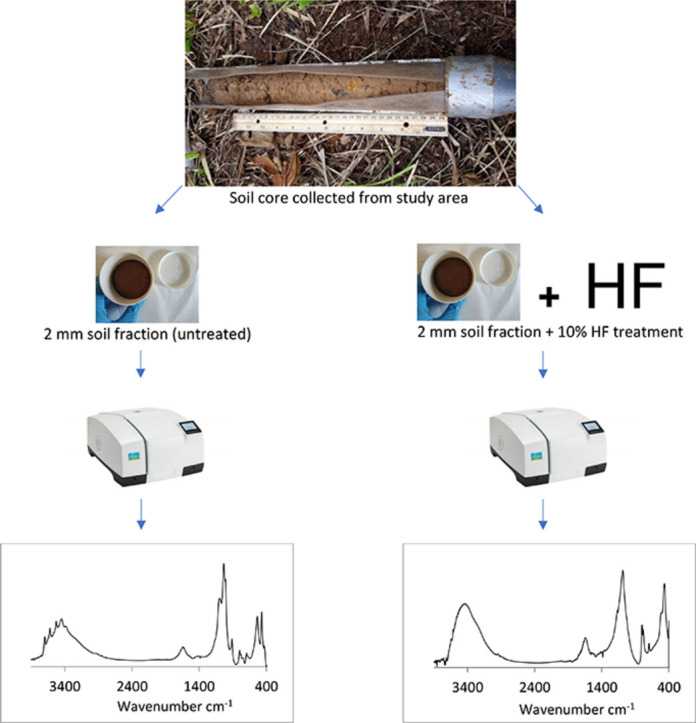

Specification Table

**Table:** 

Subject Area:	Environmental Science
More specific subject area:	Soil carbon biogeochemistry
Method name:	HF-treatment of mineral soils for FT-IR analysis
Name and reference of original method:	The procedures for the HF-treatment and FT-IR spectroscopy analyses of soil samples were modified from [4] and [7].
Resource availability:	Not applicable

## Introduction

Soils are defined as highly heterogeneous, complex and dynamic systems where soil minerals and biogenic products derivatives of microbes, roots, and plant materials at different stages of decomposition are in close vicinities and interact with each other [1]. Considering the role of soil carbon in the regulation of global climate, analytical approaches aimed at refining our understanding of the long-term dynamics of the soil carbon pool remain high on the list of research priorities. In soils, the stability of organic carbon is a not only a function of molecular structure [2], but more importantly of their interactions with secondary minerals and pedogenic oxides [3,4]. However, due to the complex nature of soils, many studies regarding organo-mineral interactions have narrowed their focus to the use of quantitative descriptors, such as isotherm shapes to yield empirical relationships from which soil carbon dynamics are inferred [5]. On the other hand, only a limited number of studies have focused on a more direct approach using incubation experiments [3].

In this contribution, we combined HF-treatment, FT-IR spectroscopy, powdered X-ray diffraction (XRD), and energy dispersive X-ray fluorescence (ED-XRF) as a direct approach to provide further insights into the interactions between SOM and secondary minerals in situ. Pre-treating the mineral soils with a 10% HF solution concentrates the OM in the HF-treated soil as well as removes metal species [6]. By concentrating the OM, this improves spectral resolution of the samples, while the removal of metallic species provides us with an opportunity to broaden our understanding of the role of inorganic soil variables in the adsorption and physical protection of SOM. It is important to note that HF-treated soils are generally analyzed by NMR spectroscopy, and that treatment with HF does not alter the overall OM composition of the soil [7,8]. Infrared spectroscopy has evolved as a powerful analytical technique in soil carbon biogeochemistry research as it is capable of providing well resolved spectral information that is often a prerequisite for the elucidation of complex organo-mineral interactions. This is evidenced by its use in the structural elucidation and quantification of biological samples, as well as environmental matrices such as SOM, and compost [9], [10], [11]. Critically, FT-IR provides us with a rapid, non-destructive means to determine which organic structures preferentially associate with clay mineral surfaces, which may be accessible to decomposers, and which are physically protected from decomposition at the molecular scale. Furthermore, in IR spectroscopy, samples are not subjected to chemical treatments thus averting secondary reactions; and all compounds present in a sample are measured simultaneously, thereby simplifying and expediting the analytical process. Additionally, the content of primary biomacromolecules carbohydrates, proteins, and fats can be determined quantitatively from a single spectrum [12]. When considered with XRD and ED-XRF, this provides us with the ability to further decipher complex organo-mineral interactions.

## Method details

### Methodology

#### Site description and sample collection

**Fig. 1:**
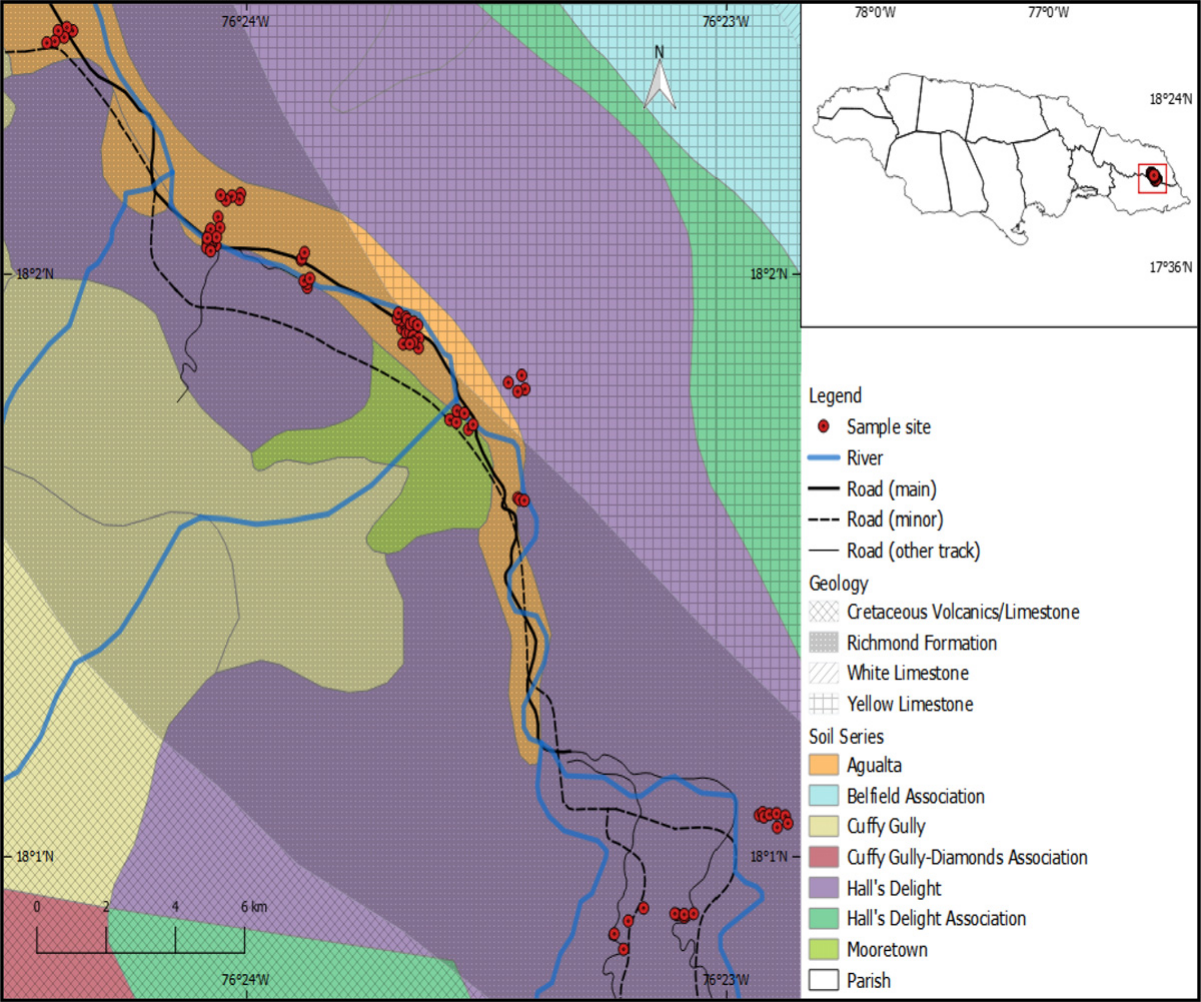
Distribution of soil sample collection sites (stratified according to soil series and geology) in the Millbank farming region, Upper Rio Grande Valley, Jamaica. (For interpretation of the references to color in this figure legend, the reader is referred to the web version of this article.)

Composite soil samples (0–30 cm) reflecting different land-use types were collected using an Edelman hand auger (January - August 2019) from various sites along a transect (5.9 km, of an area encompassing 340 ha) [13]. The sampling area was dominated by *Colocasia esculenta* (dasheen) cultivation in the northwest and a forest reserve in the southeast, in the adjoining rural communities of the Upper Rio Grande Valley (18.04°N, 76.40°W). The valley is flanked by Cretaceous rocks ascending to the Main Ridge of the Blue Mountains in the southwest, and by Palaeocene limestone escarpment of the John Crow Mountains in the northeast [14] (Fig. 1). The Highland soil of the valley may be further divided into 1) inceptisols—generally immature, highly porous, acidic, and low in nutrients except in forested areas which allows for the development of an organic-rich upper layer; and 2) ultisols and vertisols—less acidic clay-rich soils produced by the weathering of shales in areas with limited draining [15]. The local soil classification is dominated by the Hall's Delight series with a measured textural composition of 19.94% sand, 56.65% silt, and 23.86% clay: and an average bulk density of 0.94 g cm^−3^ for managed land and 0.75 g cm^−3^ for unmanaged land. Although the study area transitioned from a natural ecosystem to one dominated by anthropogenic influences, the climate along the transect is indistinguishable (source: Meteorological Services of Jamaica 2000).

#### Sample preparation

Composite soil samples were air-dried, disaggregated, and all debris (e.g., plant material and stones) removed before being sieved through a stainless-steel sieve with a 2-mm aperture. A subsample of the <2 mm soil fraction was ground in an agate mortar, and then sieved through a 150 μm stainless steel sieve. The two size fractions (<2 mm and <150 μm) were stored in acid-washed screw-cap polyethylene jars before analysis. Molecular-level information was determined using the <2 mm fraction, while the <150 μm fraction was used for other analyses [13].

#### HF treatment

Approximately 5 g of the <2 mm soil fraction was transferred to a 50 mL Teflon vial and 30 mL of 10% (v/v) added to the tube on ice. Ice is used as a precautionary measure to quench any potentially very effervescent reaction when the HF solution is added to the soil in the tubes. Each vial was loosely capped and left to stand at room temperature overnight. After sitting overnight, the vials were securely closed before reciprocal shaking (150 strokes min^−1^) overnight. Samples were then centrifuges at 3500 rpm for 3 min and the supernatant decanted. Samples were subjected to two additional three-hour washes with centrifugation. After washing, samples were rinsed with copious amounts of deionized water and centrifugation 3500 rpm for 10 min (five rinses) to remove excess salts and then allowed to be air-dried for further use [6].

#### Infrared spectroscopy

Approximately 1.0 mg of dried untreated and HF-treated soils were homogenized in 100 mg of spectroscopic-grade KBr with a refractive index of 1.559 and a particle size of 5–20 µm (Sigma) and used to create a disk-like KBr pellet for analysis. Spectra were recorded on a Perkin Elmer FT-IR Spectrum GX Spectrometer by accumulating 256 scans (to increase the signal-to-noise ratio) in the 4000 to 400 cm^–1^ mid-infrared spectral range in the absorbance mode with a resolution of 4 cm^–1^. Background KBr spectra were obtained and all spectra ratioed to the background. Baseline corrections for all spectra were done using the automatic baseline correction method. Samples were analyzed immediately after preparation to minimize the suppression of key signals by KBr-adsorbed atmospheric water [3].

#### Powdered X-ay diffraction

Powdered XRD analysis of untreated and HF-treated soils were performed on a Bruker AXS D8 Advanced diffractometer with collimated nickel-filtered Cu-K_α_ radiation (0.1543 nm), at 40 kV and 40 mA. A Bruker DEFRAC Evaluation Data Collector and an EVA Graphics and Identification software packages were employed to capture and process raw data. Scans were carried out at a speed of 1°2θ/min and increments of 0.02 ([°2θ]; [3].

#### Energy dispersive X-ray fluorescence

Approximately 4 g of dried untreated and HF-treated soils, were homogenized with 2–3 drops of polyvinyl alcohol binder and pressed into 25 mm pellets [16]. Spectra were acquired for each pellet on a Shimadzu EDX-7000 Energy Dispersive X-Ray Fluorescence (ED-XRF) Spectrometer for 200 s with a tube voltage of 15 kV and an automatically selected current to achieve a 30% dead time. A titanium filter was used to analyze aluminum, calcium, and silicon and an aluminum filter used for magnesium under vacuum conditions, while iron was analyzed in air using a molybdenum filter, to enhance sensitivity. A soil reference material (NIST 2711) was also analyzed for quality control.

## Results

**Fig. 2:**
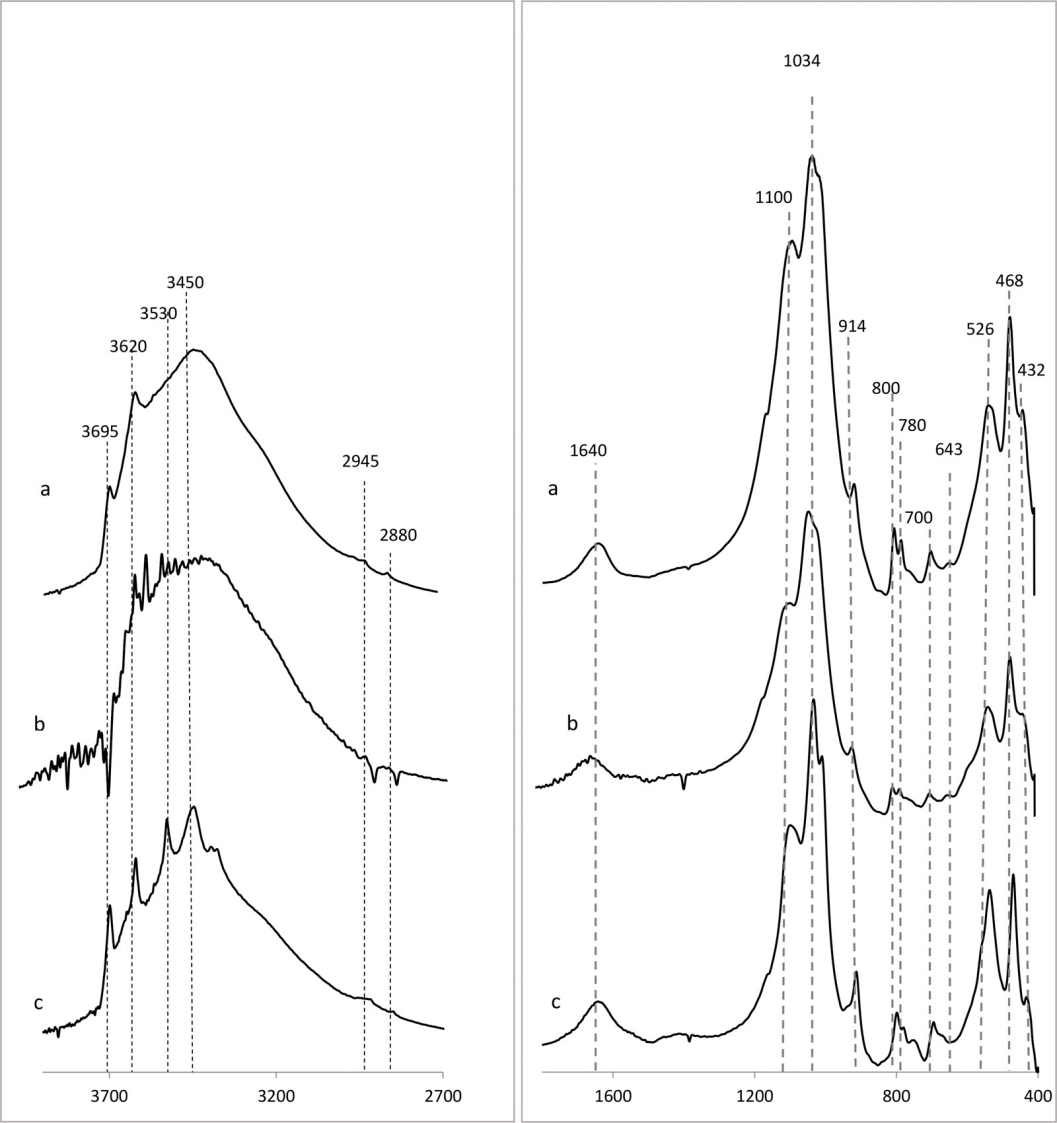
Representative infrared spectra of untreated mineral soils of the Upper Rio Grande Valley illustrating a range of polymeric molecules and organo-mineral interactions in soil of varying concentrations of OM a) low (7.6%); b) intermediate (12.3%); and c) high (21.0%). Only spectral regions of interest are displayed; 2700–3900 cm^−1^ (OH stretching region) and 1600–400 cm^−1^, (fingerprint region), and spectral assignments are presented in Table 1.

**Fig. 3:**
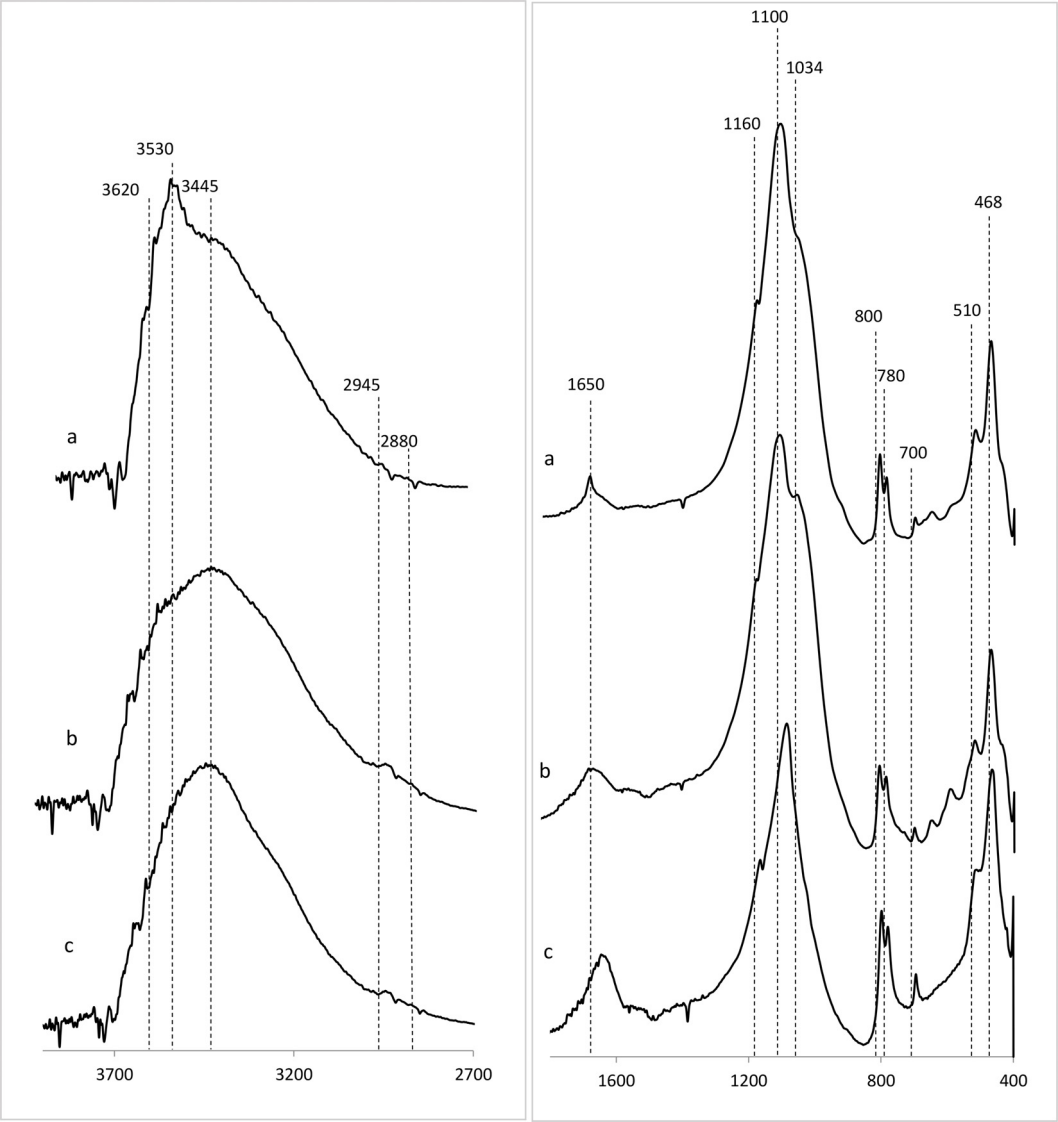
Representative infrared spectra of HF-treated mineral soils of the Upper Rio Grande Valley denoting varying concentrations of OM a) low (7.6%); b) intermediate (12.3%); and c) high (21.0%). After HF-treatment, it is now apparent that the highly overlapping OH stretching region is now characterized by more defined peaks with improved resolution and lower baselines suggesting that the organic component are concentrated and more dissociated. On the other hand, the fingerprint region of the HF-treated spectra is characterized by several nuances, which together provide further evidence of the role of inorganic mineral components in the adsorption and stabilization of OM in soils. For instance, the band at 1092 cm^−1^ assigned to signals from carbohydrates is now enriched relative to the peak positioned 1034 cm^−1^ ν(Si—O) in soil mineral, confirming a prominent role for crystalline versus amorphous silicate structures in organo-mineral interactions in soils. Additionally, diagnostic clay mineral peaks (e.g., 3965 cm^−1^ [kaolinite) and 3620 cm^−1^ [montmorillonite]) have been perturbed. Alternatively, interactions at this scale are generally only observed in incubation studies using pure clay minerals [3,4].

Soil organic matter and its interactions with soil inorganic components (e.g., minerals) represent a broad range of chemical compounds characterized by complex multiscale interactions. Therefore, some functional groups and interactions may be missed due to significant spectral overlaps typified by very broad absorbance peaks often associated with weak shoulders. As a convention, spectral assignments best define the predominant species within a given region of a particular spectrum (Figs. 2 and 3; Table 1).

**Table 1:** Positions and assignments of adsorption bands (cm^−1^) observed in the infrared spectra of composite soil samples.

Observed wavenumber (cm^1^)	Assignments
3695 3620	ν(OH) assigned to surface hydroxyl groups of kaolinite clay mineral ν(OH) of phenolic, carboxylic group, and/or carbohydrates (also O—H stretching in clay minerals) ν(OH) of hydroxyl groups coordinated to octahedral cations in clay minerals (e.g., kaolinite and montmorillonite ν(H2014O—H) and ν_as_(H—O—H) of H-bonded to water in clay minerals ν(N—H) of amide A in proteins
3445	ν(N—H) of amide B ν_as_(O—H) of H-bonded phenolic groups
2929	ν_as_(C—H) of CH_2_ groups in aliphatic structures
2856	ν(C—H) of CH_2_ groups in aliphatic structures
1640	ν(C=C) in aromatic compounds ν_as_ (O—C=O) of metal coordinated carboxylates *v*(C = O) of H-bonded amides and amide I band in peptides/proteins
1099–1095	ν(C—C), ν(C—O), ν(C—O—C) predominantly from ring vibration of carbohydrates ν(P—O) of nucleic acid
1034	ν(Si—O) of quartz (sand) ν(C—O) in polysaccharides and nucleic acids
990–500	δ(=C—H) or aromatic
914	δ(OH) of structural OH in clay minerals δ(Al-Al- OH)-)
755–700	ν(OH) surface hydroxyl groups in clay minerals
800–780	ν(Si—O)
544/526/512	δ(Si—O—Al)
468	ν(Si—O—Si)

ν- Symmetric stretching vibration.

ν_as_- Asymmetric stretching vibration.

δ – Bending vibration.

**Fig. 4:**
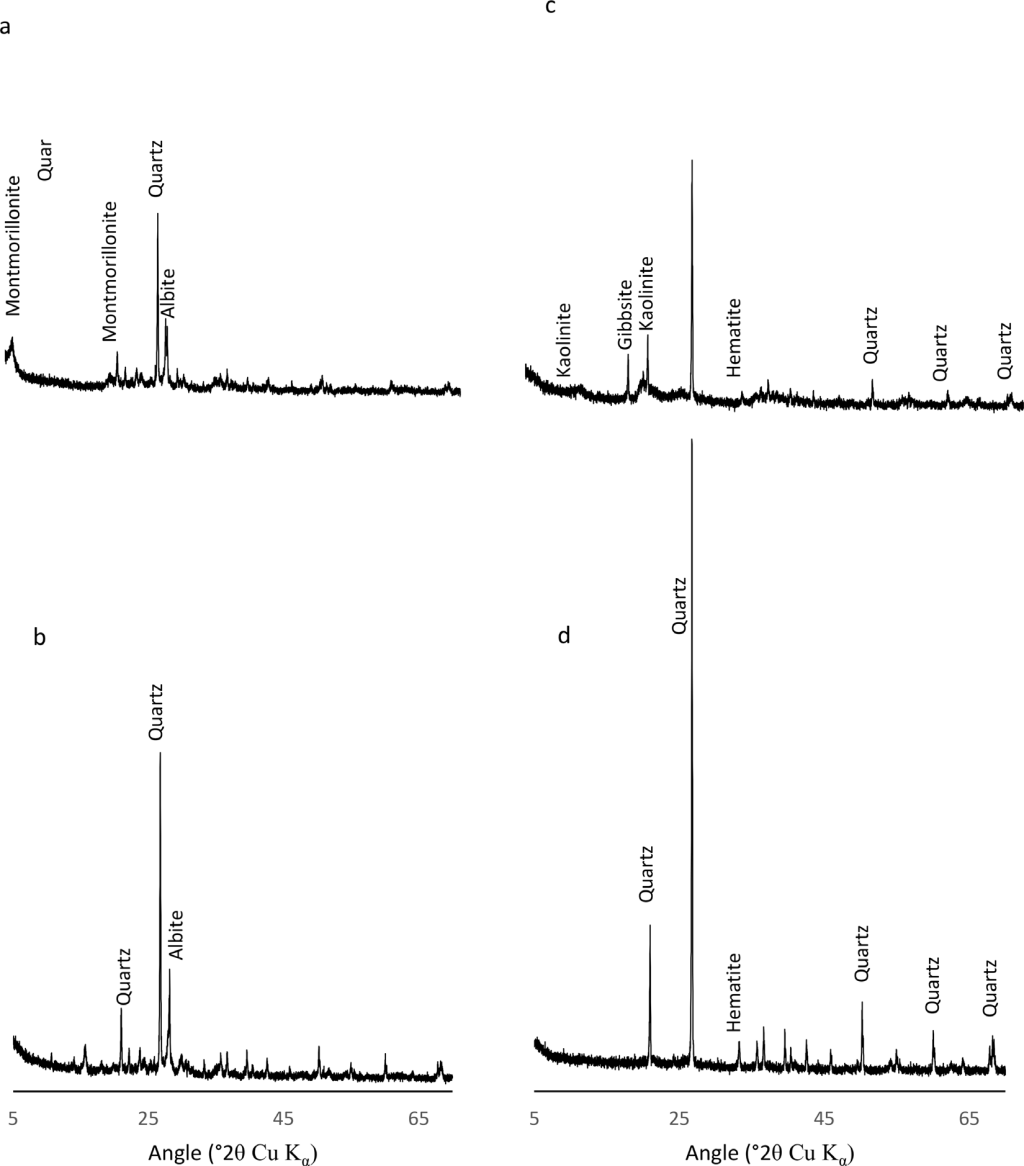
X-ray diffraction patterns for untreated (a) and 10% HF-treated (b) mineral soils of low OM content (7.6%) and untreated (c) and 10% HF-treated (d) samples of high OM (21%) content.

To further unravel the complexities of soil organo-mineral interactions, here we also employ powdered X-ray diffraction to probe the effect of the 10% HF solution on soil minerals and the representative diffraction patterns presented in Fig. 4. The diffraction patterns of the untreated soil samples clearly illustrate the presence of ubiquitous phyllosilicate minerals typified by montmorillonite (Fig. 4a); in the low OM soil), and kaolinite and gibbsite (Fig. 4c; in the high OM soil). However, after HF-treatment has occurred, the representative peaks have now disappeared, and signals commonly assigned to quarts, hematite, and albite appear relatively enriched (Fig. 4b, d). This is not surprising, as quartz and albite (a feldspar mineral) have been shown to be more resistant to Hf-treatment, relative to most silicate minerals [17]. Additionally, the preservation of hematite (Fe_2_O_3_) in the HF treated sample, can be attributed to the presence of ferric ion species, which are innately resistant to HF. Hydrofluoric acid removes ferrous minerals by binding to the ligand site in the Fe^2+^complex; however, this binding is not facilitated at Fe^3+^ sites [18], [19], [20].

**Fig. 5:**
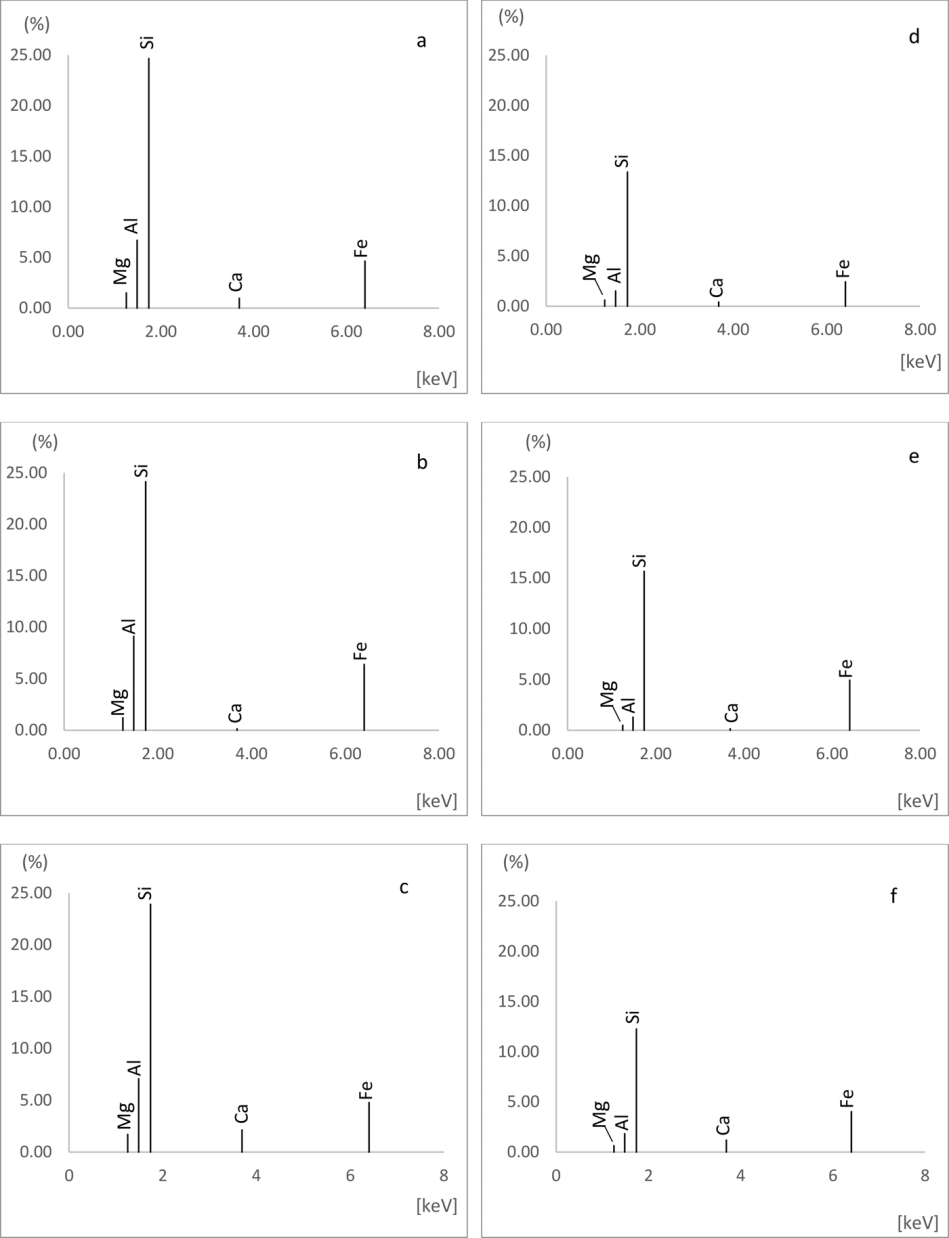
Concentrations of metals of interest in untreated (a–c) and HF-treated soils (d–f) soils of low (a, d); medium (d, e); and high (c, f) OM contents.

In order to better understand the spatial co-variations of SOM with soil minerals, we apply ED-XRF, which allows us to disentangle complex soil minerals and navigate the dynamics of specific metals (Si, Al, Mg, Ca, and Fe) in solid-state, before and after HF-treatment (Fig. 5). Further, the concentrations of the same suite of metals in the HF digest were determined by the mass balance. The concentrations of the metals of interest in the untreated and HF-treated soils are summarized in Table 2 and are generally statistically different (*p* *=* *0.05*), except for that of Ca. The liability of these metals in mineral acid has previously been shown [4].

**Table 2:** Summary table with the average values for Al, Ca, Fe, Mg, and Si in the untreated and HF-treated soil samples.

Element	Concentration of elements as determined by XRF (%)	
	*Untreated*	*HF-Treated*
Al	8.18 ± 1.31	1.34 ± 0.45
Ca	0.64 ± 0.69	0.42 ± 0.35
Fe	6.14 ± 1.43	4.87 ± 1.69
Mg	1.30 ± 0.30	0.54 ± 0.09
Si	23.39 ± 2.45	14.18 ± 1.71

**Fig. 6:**
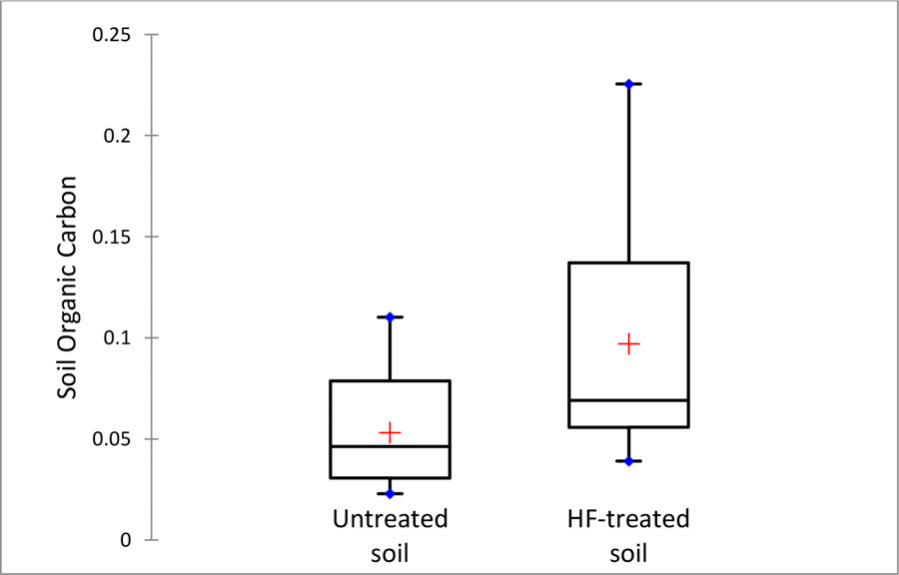
Soil organic carbon determined by loss on ignition and represented as the fraction of organic matter per one gram of HF-treated and untreated soils. (For interpretation of the references to color in this figure legend, the reader is referred to the web version of this article.)

**Fig. 7:**
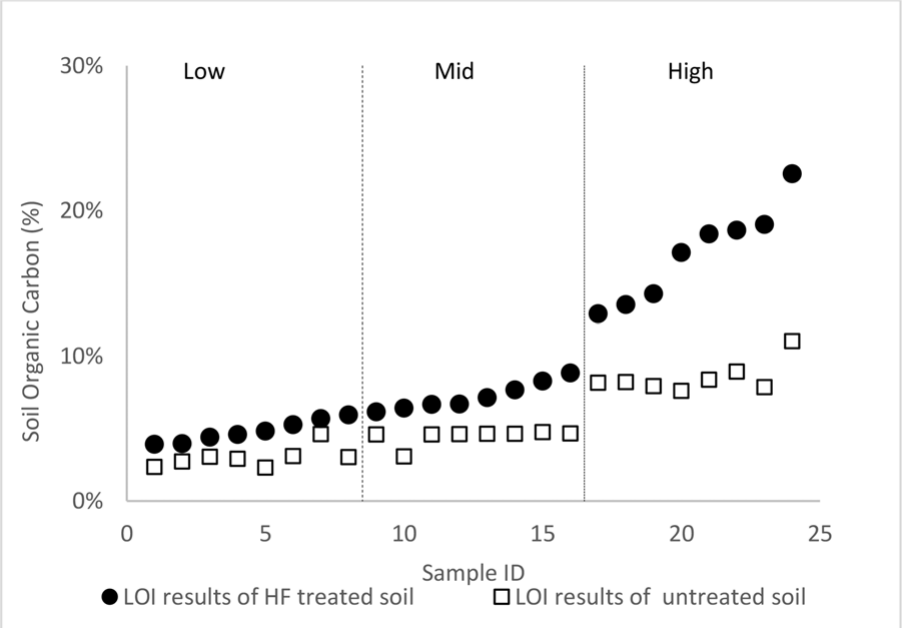
Comparison of SOC content before and after HF- treatment. Here the sample population is stratified according to their relative OM content.

Additionally, we determine the concentrations of organic carbon in the untreated and HF-treated soil samples via dry combustion [21] and the aggregate results presented in Fig. 6. The OM content varied widely in both untreated (6.03% to 29.00%) and HF-treated (6.73% to 38.89%) soils. In general, there is no statistical significance (*p* *=* *0.05*) between the OM content of the untreated (13.99 ± 6.59%) and HF-treated (16.72 ± 9.90%) samples. This is expected since HF does not alter the overall OM composition of the soil; but rather concentrates it relative to other components in the matrices after the removal of metal species. We further sub-categorized the samples by their OM values to probe how the initial levels of OM in the untreated soil influence the results of the HF-treatment samples. The results suggest that the HF pre-treatment significantly concentrates the OM (29.41 ± 5.68%) in soils which were initially rich in organics (22.39 ± 2.87%) at alpha = 0.05 (Fig. 7). This difference may be due in part to the higher OM:mineral content in these soil.

## Declaration of Competing Interest

The authors declare that they have no known competing financial interests or personal relationships that could have appeared to influence the work reported in this paper.

## Data Availability

Data will be made available on request.
